# An Insight on the Possible Association between Inflammatory Bowel Disease and Biologic Therapy with IL-17 Inhibitors in Psoriasis Patients

**DOI:** 10.3390/pharmaceutics15082171

**Published:** 2023-08-21

**Authors:** Olguța Anca Orzan, Cristian George Țieranu, Andrei Ovidiu Olteanu, Alexandra Maria Dorobanțu, Anca Cojocaru, Mara Mădălina Mihai, Liliana Gabriela Popa, Ana Maria Gheorghiu, Călin Giurcăneanu, Ana Ion

**Affiliations:** 1 Department of Oncologic Dermatology, ‘Carol Davila’ University of Medicine and Pharmacy, 020021 Bucharest, Romania; olguta.orzan@umfcd.ro (O.A.O.); liliana.popa@umfcd.ro (L.G.P.); calin.giurcaneanu@umfcd.ro (C.G.); 2 Department of Dermatology, ‘Elias’ University Emergency Hospital, 011461 Bucharest, Romania; alexandramdorobantu@gmail.com (A.M.D.); anka_cojocaru@yahoo.com (A.C.); anaion00@yahoo.com (A.I.); 3 Department of Gastroenterology, ‘Carol Davila’ University of Medicine and Pharmacy, 020021 Bucharest, Romania; dr.olteanuandrei@gmail.com; 4 Department of Gastroenterology, ‘Elias’ Emergency University Hospital, 011461 Bucharest, Romania; 5 Department of Rheumatology, ‘Carol Davila’ University of Medicine and Pharmacy, 020021 Bucharest, Romania; ana-maria.gheorghiu@umfcd.ro; 6 Internal Medicine and Rheumatology, ‘Cantacuzino’ Hospital, 011438 Bucharest, Romania

**Keywords:** IL-17 inhibitor adverse effects, interleukin 17 inhibition inflammatory bowel disease, psoriasis therapy adverse events, multidisciplinary approach psoriasis

## Abstract

Psoriasis is a chronic, inflammatory, multisystemic disease which affects approximately 2–3% of the population globally, whose onset is triggered by genetic and environmental factors which activate both dendritic cells and keratinocytes, resulting in the production of proinflammatory cytokines such as tumor necrosis factor alpha, interleukin 17, interleukin 23, interleukin 22, and interleukin 1β. An in-depth understanding of the pathophysiology of psoriasis led to significant advances in the development of safe and efficient novel therapeutic options, with four classes of biologic therapy being approved for the management of moderate to severe psoriasis: tumor necrosis factor alpha inhibitors, interleukin 23 inhibitors, anti-interleukin 12/23 agents, anti-interleukin 17 agents, as well as small-molecule inhibitors, such as apremilast. Psoriasis is associated with comorbid conditions, namely psoriatic arthritis, cardiovascular disease, metabolic syndrome, psychiatric disorders, malignancy, as well as inflammatory bowel disease. For patients affected by both psoriasis and inflammatory bowel disease, there is a strong recommendation to avoid IL-17 inhibitors since they may play a part in the exacerbation of the gastrointestinal disease. Our aim was to perform a thorough literature review regarding the development of inflammatory bowel disease lesions in psoriasis patients treated with IL-17 inhibitors, along with a case presentation to emphasize the need for close follow-up of these patients.

## 1. Introduction

Psoriasis is a chronic, inflammatory, multisystemic disease which affects approximately 2–3% of the population globally [1]. Its onset is triggered by genetic and environmental factors whose close interplay predisposes individuals to the psoriasis phenotype [2]. Genetic factors refer especially to the PSORS 1 interval located on the short arm of the chromosome 6p21.3, which contains the allele HLA-Cw6, which further encodes a class I major histocompatibility complex that mediates activation and proinflammatory response of the T cells [3]. The main environmental factors responsible for the triggering of the disease consist in medication (beta blockers, lithium), physical trauma, and infections (especially streptococcal infections) [2]. Injury due to mechanical trauma leads to the release of cathelicidin by keratinocytes, which forms complexes with either self-produced DNA or pathogen-derived DNA, which further activate plasmacytoid dendritic cells [4]. This promotes T-cell-mediated inflammation through the activation of local myeloid dendritic cells by tumor necrosis factor alpha (TNF-alpha), interleukin (IL) 6, and IL (IL) 1β [4]. Moreover, in 2014, Lande R et al. found that cathelicidin (LL37) may play a part in directly activating auto-reactive circulating T lymphocytes, a phenomenon particularly seen in psoriasis patients with a greater disease activity [5]. Myeloid dendritic cells have the ability to migrate into the local lymph nodes and secrete proinflammatory cytokines, such as TNF-alpha, interleukin (IL) 12, and interleukin (IL) 23, which activate T lymphocytes [2]. T cells further migrate towards the site of skin inflammation and produce effector molecules that activate keratinocytes, which results in a cascade of cytokines and chemokines that continue to activate inflammatory cells, the main proinflammatory cytokines found in psoriasis being TNF-alpha, IL–17, IL-23, IL-22, and IL-1β [2]. This succession of inflammatory events translates clinically into well-demarcated, round to oval, erythematous plaques covered by white, silvery scales, usually occurring in a symmetrical distribution on the elbows, knees, scalp, and trunk [2].

Among the main cells responsible for the production of chemokines and cytokines are keratinocytes and dendritic cells [2]. It is believed that keratinocytes are essential in the pathogenesis of psoriasis during the early stage, as well as later on in the course of the disease [6]. Injury to the skin leads to the release of antimicrobial peptides such as LL37, β-defensins, and S 100 proteins, which enhance the upregulation of IL-6 and IL-10, as well as CXCL8 and CXCL10, which further stimulates the recruitment of macrophages and neutrophils [2]. Apart from being an important source of antimicrobial peptides, keratinocytes also release TNF-alpha, IL-18, and IL-1β, which promote differentiation of T helper 1 and T helper 17 cells [7]. The activation of T helper 1 and T helper 17 cells leads to the release of IL-22 and IL-17, respectively, which drives the proliferation and activation of keratinocytes, thus setting up a positive feedback loop [7]. Keratinocytes are also responsible for the production and release of vascular endothelial growth factor (VEGF), which enhances angiogenesis, leading to the erythematous appearance of the psoriasis plaque [8]. Dendritic cells, as antigen-presenting cells, are an important source of cytokines in psoriasis [2]. Two lineages of dendritic cells participate in the proinflammatory cascade found in psoriasis: plasmacytoid dendritic cells and myeloid dendritic cells [9]. Plasmacytoid dendritic cells release abundant quantities of type 1 interferon, an archetypal cytokine in psoriasis whose levels are increased in lesional skin compared to normal skin [10]. Plasmacytoid dendritic cells are activated by the LL37-DNA complexes and contribute to the inflammatory events from the very early stages of psoriasis [10]. Neutrophils are believed to be important through recruitment and activation of T lymphocytes and the proliferation of keratinocytes [11]. In 2015, Reich K et al. found that there might by a neutrophil–keratinocyte cross-talk which takes place early in the pathogenesis of psoriasis [12]. It seemed that secukinumab, an IL-17 inhibitor, had the ability to reduce the level of cutaneous neutrophils while improving the histological aspect of keratinocytes and stratum corneum in the lesional skin [12]. There is a key role for T lymphocytes in the pathophysiology of psoriasis indicated by their significant prevalence in skin biopsies [13]. The subsets of T lymphocytes intensely studied in psoriasis are CD4+ T helper 1, T helper 17, and T helper 22, which secrete TNFα/IFNγ, IL-17/Il-22, as well as IL-22, respectively [14]. It is considered that T lymphocytes undergo an abnormal activation in psoriasis, particularly with the IL-23/T helper 17 axis being crucial to the intertwined process of the disease [15].

A central point in the pathogenesis of psoriasis is represented by the increased amount of cytokines and chemokines [9]. TNF-alpha is a major cytokine produced in the context of cutaneous inflammation by numerous cells: T helper 1 cells, T helper 17 cells, T helper 22 cells, keratinocytes, and macrophages [16]. TNF-alpha exerts a wide range of effects; it enhances the expression of C reactive protein, as well as that of several cytokines, such as IL-6 and IL-8, while also facilitating the production and release of IL-23 and IL-17 [9]. Even though TNF-alpha inhibitors are an appropriate therapeutic option, the adverse effects of TNF-alpha blockade favor, nowadays, the use of other biologic therapy classes [2]. IFNγ is another relevant cytokine in the physiopathology of psoriasis [17]. T helper 1 cells, natural killer cells, and dendritic cells are a major source of IFNγ [17]. It has been found that IFNγ promotes the release of IL-23 and IL-1, which, in turn, promote the differentiation and activation of T helper 17 and T helper 22 lymphocytes [18].

IL-23, a cytokine released by dendritic cells and macrophages, is involved in mediating the terminal differentiation and, furthermore, the activation of T helper 17 cells, upregulation of TNF-alpha expression in macrophages, and activation of keratinocytes [19]. Since it has been found that psoriasis lesions have an increased amount of IL-23, its inhibition with anti-IL-12/23 and anti-IL-23 agents has proven to be highly effective [20].

**Figure 1:**
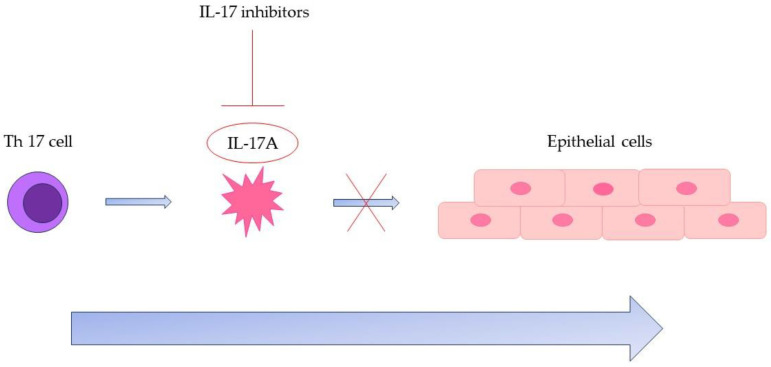
The effect of IL-17 inhibitors on the proinflammatory cascade involving Th 17 cells and IL-17A. Abbreviations: Th 17 cell—T helper 17 cell; IL-17—Interleukin 17.

IL-17A is a proinflammatory cytokine which pertains to the IL-17 A-F family [21]. In a clinical trial from 2012, Krueger JG et al. demonstrated the undeniable and, at the present moment, widely recognized involvement of IL-17 A in the immunopathogenesis of psoriasis [22]. In the study, skin lesions from 40 psoriasis patients who were participating in a randomized, double-blind, and placebo-controlled trial of ixekizumab, an IL-17 inhibitor, in which they received 5 mg, 15 mg, 50 mg, and 150 mg ixekizumab or placebo at baseline and at weeks 2 and 4 were studied [22]. In terms of histologic changes, the authors found a dose-dependent reduction in keratinocyte proliferation, acanthosis, and overall epidermal hyperplasia, as well as in the dermal and epidermal infiltration with T cells and dendritic cells at 2 weeks [22]. By week 6, the skin appeared normal [22]. IL-17A is produced and released by neutrophils, natural killer cells, mast cells, and T helper 17 cells, and its receptors are found mostly on the keratinocytes [21]. A visual representation of the mechanism of action of IL-17 inhibitors can be seen below in Figure 1.

In a comparative study from 2006 by Liang SC et al. on the coexpression of IL-17 and IL-22 by T helper 17 cells, the authors showed that IL-17A has a pronounced and direct effect on the regulation of genes found on keratinocytes which are strongly involved in the innate defense such as LL37/cathelicidin, S100 family proteins, defensins, lipocalin, as well as a range of chemokines which regulate neutrophil circulation [23]. Once stimulated by IL-17A, keratinocytes produce antimicrobial peptides, chemokines such as IL-8, and cytokines such as IL-1, IL-6, IL-23, and IL-19 [24]. Moreover, IL-17A facilitates the cutaneous recruitment of both T cells and dendritic cells [25]. The pronounced elevation of TNF-alpha, IL-17, IL-23, IL-22, and IL-1 β promotes an overall systemic chronic subclinical inflammation, which, along with shared genetic features, explains the association between psoriasis and other comorbid conditions, namely psoriatic arthritis (PsA), cardiovascular disease, metabolic syndrome, psychiatric disorders, malignancy, as well as inflammatory bowel disease (IBD) [2].

An in-depth understanding of the pathophysiology of psoriasis led to significant advances in the development of safe and efficient novel therapeutic options, with four classes of biologic therapy being approved for the management of moderate to severe psoriasis: TNF-α inhibitors, anti-IL-23 agents, anti-IL-12/23 agents, IL-17 inhibitors, as well as small-molecule inhibitors, such as apremilast, as presented in Table 1 [26].

Nevertheless, in many instances, the concomitant diseases associated with psoriasis greatly impact the clinical decision of selecting one therapeutic class over another [26,27]. Therefore, when planning an individualized therapeutic approach, it is recommended to carefully take into consideration which biologic agent improves or exacerbates a certain comorbidity for each patient, as presented in Table 2 [26].

For patients with psoriasis and psoriatic arthritis, first-line therapy is represented by TNF-α inhibitors, ixekizumab, secukinumab, guselkumab, and risankizumab [26]. Patients with both psoriasis and congestive heart failure should be treated with IL-17 and IL-23 inhibitors with the recommendation to avoid TNF-α inhibitors in NYHA class III or IV [26]. In cases with concomitant psoriasis and metabolic syndrome, IL-17 inhibitors, ustekinumab, IL-23 inhibitors, and apremilast are recommended [26]. For patients affected by psoriasis and depression, first-line therapy is represented by IL-23 inhibitors [26]. If malignancy is concomitant with psoriasis, TNF-alpha inhibitors should be avoided, while IL-17 inhibitors as well as IL-23 inhibitors are more suitable options [26]. Finally, for patients affected by both psoriasis and IBD, first-line therapy is represented by TNF-alpha inhibitors, particularly adalimumab and infliximab, with second-line therapy involving certolizumab pegol, ustekinumab, and IL-23 inhibitors, with the strong recommendation to avoid IL-17 inhibitors [26]. By studying case reports on the development of inflammatory bowel disease lesions in psoriasis patients treated with IL-17 inhibitors, along with a case presentation, we aim at increasing the awareness of the need for close follow-up of these patients.

## 2. Materials and Methods

An in-depth literature review regarding the implications of IL-17 inhibitors used for the management of psoriatic disease in the onset of inflammatory bowel disease was developed. The gastrointestinal adverse effects of IL-17 inhibitors have been reported in randomized clinical trials with ixekizumab and secukinumab. Nevertheless, in this paper, we highlighted the gastroenterologic adverse reactions reported post-marketing, in psoriasis patients under biologic therapy with an IL-17 inhibitor.

We aimed at finding case presentations that reported the appearance or aggravation of inflammatory bowel disease in the context of biologic therapy with IL-17 inhibitors for psoriasis. The search was performed through PubMed as the main electronic database. The following keywords were used: “interleukin 17 inhibition inflammatory bowel disease”, “IL-17 inhibitors adverse effects”, “psoriasis therapy adverse events”, “multidisciplinary approach psoriasis”. We included case reports from 2018 to 2021.

Data on patient characteristics concerning their age, sex, personal and family history, previous treatments for psoriasis, and their potential comorbid conditions were selected. Moreover, information regarding the type of biologic agent used for psoriasis, their gastrointestinal symptoms on presentation, the investigations carried out, the final diagnosis, and management of the newly onset inflammatory bowel disease was extracted. The gathered data were displayed in a table in order to adequately emphasize the patient characteristics and further management for each case report.

Furthermore, we brought the experience of our dermatology department by documenting the case of a 33-year-old male patient with a long-standing history of psoriasis under biologic therapy, previously treated with methotrexate. During therapy with methotrexate, the patient mentioned mild, unspecified gastrointestinal symptoms which persisted after switching to biologic therapy. The patient correlated these symptoms with poor dietary habits. Nevertheless, further investigations led to the diagnosis of Crohn’s-like ileitis, possibly in the context of biologic therapy with an IL-17 inhibitor.

## 3. Influence of Biologic Therapy on the Course of Inflammatory Bowel Disease

Both psoriasis and IBD are inflammatory diseases with a chronic course marked by relapses, as well as periods of clinical inactivity [28]. In recent years, epidemiological studies have been able to establish that there is, indeed, a strong association between psoriasis and IBD [29]. A meta-analysis from 2020 by Alinaghi F et al. concerning the global prevalence and bidirectional association between psoriasis and IBD shed light on the interconnection between the two chronic inflammatory diseases [29]. The metanalysis included 93 studies starting from April 2018 which reported data on chronic plaque psoriasis among patients with IBD, as well as vice versa, from the PubMed, Web of Science, and EMBASE databases [29]. A meta-analysis was performed to estimate the prevalence and associations between IBD and psoriasis [29]. Results showed that the prevalence of psoriasis was 3.6% for patients with Crohn’s disease (CD) and 2.8% for patients with ulcerative colitis (UC) [29]. The prevalence of CD in patients affected by psoriasis was 0.7%, while the prevalence of UC was 0.5% [29]. The presence of either CD or ulcerative colitis was significantly associated with psoriasis, with an odds ratio of 2.0 and 1.5, respectively [29]. The presence of psoriasis was, as well, significantly associated with ulcerative colitis (odds ratio 1.6) and CD (odds ratio 2.2) [29]. Therefore, a bidirectional co-occurrence between IBD and psoriasis was found, increasing acknowledgement among physicians in the diagnostic and therapeutic process of both the cutaneous and gastrointestinal diseases [29]

A systematic review and meta-analysis by Fu Y, Lee C-H, and Chi C-C from 2018 regarding the association between IBD and psoriasis which included five cross-sectional studies and four cohort studies with 7,794,087 participants found a significant link between psoriasis and CD (odds ratio (OR) 1.70) and between psoriasis and UC (odds ratio 1.75) [28]. Moreover, patients with psoriasis had an elevated risk of CD (risk ratio 2.53) as well as UC (risk ratio 1.71) [28]. Because of the shared genetic and immunologic features of psoriasis and IBD, certain classes of biologic therapy are nowadays used to control flares for both diseases, especially TNF-alpha inhibitors, ustekinumab, and IL-23 inhibitors [26,30,31,32,33,34,35].

Regarding TNF-alpha inhibitors, adalimumab and infliximab show similar effectiveness in both CD and UC; therefore, they could be used as first-line therapy, while certolizumab pegol, a biologic agent with a more reduced effectiveness in CD, should be used as second-line therapy [26]. A large cohort study from 2019 by Korzenik J et al., which included 17,018 participants under anti-TNF alpha agents for other autoimmune diseases other than IBD, aimed to assess the risk of developing either CD or UC [36]. Results showed that only patients under etanercept had an increased risk of being equally affected by either UC or CD, with a hazard ratio (HR) of 2.0 for both diseases [36]. In 2007, Ahmad K and Rogers also reported a case of Crohn’s disease in a patient under etanercept for psoriasis [37]. The patient was a 68-year-old male with long-standing history of psoriasis previously treated with methotrexate, etretinate, and ciclosporin, which was withdrawn due to renal impairment [37]. Consequently, biologic therapy with infliximab, a TNF-alpha inhibitor, was initiated, with an excellent response [37]. Nevertheless, after four months of biologic therapy with infliximab, the patient developed severe neutropenia requiring therapy with granulocyte colony stimulating factor [37]. Accordingly, the patient was switched to etanercept, 25 mg every two weeks, with an adequate control of the cutaneous disease [37]. Six months later, the patient presented with abdominal pain with 10 episodes of diarrhea daily [37]. Colonoscopy revealed friable mucosa, decreased vascular pattern, and marked inflammation [37]. Histology showed cryptitis, cryptic abscesses, as well as granuloma formation, consistent with CD [37]. Biologic therapy with etanercept was ceased and the patient was switched to adalimumab, 40 mg fortnightly, the intestinal symptoms being significantly reduced after three months [37]. Consequently, an excellent control of both psoriasis and CD was obtained [37]. In 2014, Tichy M and Hercogova J reported the case of a 43-year-old woman with psoriasis under treatment with etanercept who, after 24 months of therapy, developed Crohn’s disease [38]. Biologic therapy with etanercept was discontinued, and she received methylprednisolone 32 mg daily and mesalazine 4 g daily until her condition improved [38]. Maintenance therapy was represented by prednisolone 5–10 mg daily, with infliximab or adalimumab being considered more suitable options for the management of psoriasis [38].

Concerning IL-17 inhibitors, UNCOVER-2 and UNCOVER-3, two phase 3 trials which showed the efficacy and safety of ixekizumab for patients with moderate to severe psoriasis, reported four cases of CD and seven cases of UC compared to zero in the placebo-treated group [39]. In 2017, Reich K et al. published data from an integrated database of seven uncontrolled and randomized controlled trials concerning inflammatory bowel disease among psoriasis patients under ixekizumab [40]. The authors found that patients affected by psoriasis had an increased risk for developing inflammatory bowel diseases, compared to the general population; new-onset cases of UC and CD were uncommon, with a low incidence (<1%) in patients under ixekizumab [40]. Nevertheless, one limitation of the research was considered to be the lack of particular questions from the case report forms concerning patients’ personal and family history of IBD [40]. In 2019, Schreiber S et al. published a meta-analysis on the incidence of IBD in patients with psoriasis, psoriatic arthritis, and ankylosing spondylitis under biologic therapy with secukinumab [41]. Results showed that, from the 5181 patients affected by psoriasis, there were 14 cases of UC, five cases of CD, and one case of unclassified IBD [41]. Of these 20 cases, 14 were new-onset [41]. The authors concluded that, overall, cases of CD, UC, and unclassified inflammatory bowel disease were relatively uncommon among patients treated with secukinumab [41].

Results of preliminary trials concerning IL-23 inhibitors for the management of CD have proven encouraging based on the data [26,42,43]. A randomized, phase II clinical trial from 2022 by Sandborn WJ et al. on the use of guselkumab for the treatment of CD which included 309 patients showed that guselkumab induced major improvement in both clinical and endoscopic appearance at week 12 versus placebo with an adequate safety profile [44]. Likewise for concomitant psoriasis and CD, two case reports showed the efficacy of guselkumab in controlling both diseases, after a previous lack of response to biologic therapy with infliximab or ixekizumab [45,46]. FORTIFY, a phase three clinical trial from 2022 by Ferrante M et al. on the use of risankizumab for the maintenance of moderate to severe active Crohn’s disease included 542 patients, of which 179 were assigned to the risankizumab 180 mg group, 179 participants to the 360 mg risankizumab group, and 184 subjects to the placebo group [47]. Results showed that a greater clinical remission and better endoscopic outcomes were found in patients receiving 360 mg risankizumab versus placebo [47]. Higher rates of endoscopic response rates and clinical remission were also obtained in patients receiving 180 mg risankizumab versus placebo [47]. Following the FORTIFY trial, the authors concluded that maintenance therapy with risankizumab for patients with moderate and severe Crohn’s disease was efficient and well-tolerated [47].

Ustekinumab, an IL-12/23 inhibitor, is recommended as a second-line agent for patients affected by both psoriasis and inflammatory bowel disease, as many clinical studies support its efficacy in controlling both diseases with a proper safety profile [33,34,35,48,49].

In a clinical trial from 2019, Li K et al. assessed the effects of ustekinumab on the histological disease activity in patients with Crohn’s disease, given the fact that this therapeutic agent had an already established efficacy in this category of patients [34]. The study included 251 patients in phase three induction and maintenance studies [34]. For each patient, two endoscopic biopsies were taken at week 0, 8, and 44 from the ileum, splenic flexure, and rectum [34]. Based on global histology activity scores (GHASs), the histologic activity was determined through microscopic examination [34]. Therefore, results showed that, at week eight, the mean GHAS was, indeed, significantly reduced after induction therapy with ustekinumab compared to placebo [34]. At week 44, the mean GHAS remained reduced from week 8 in patients who received 90 mg ustekinumab every 8 weeks, but not in those receiving ustekinumab every 12 weeks or placebo [34]. In the randomized and nonrandomized maintenance therapy population, a continuous histologic improvement was found for subjects receiving 90 mg ustekinumab every 8 weeks versus those given ustekinumab every 12 weeks or placebo [34]. The authors concluded that there was a histologic improvement in a greater proportion of subjects receiving ustekinumab versus placebo [34]. The most significant improvement occurred in those receiving ustekinumab every eight weeks [34].

A clinical study from 2019 by Ahmed Z et al. compared the effectiveness of ustekinumab versus adalimumab in terms of the induction of remission of Crohn’s disease [35]. The study included 163 patients with Crohn’s disease, of which 97 were induced with adalimumab and 66 were assigned to ustekinumab [35]. Among the TNF-naïve patients, adalimumab was superior to ustekinumab in inducing a clinical response (69/89 patients vs. 4/10 patients), but not clinical remission [35]. Nevertheless, among TNF-experienced patients, adalimumab was inferior in inducing clinical response (2/8 patients vs. 15/56 patients) as well as clinical remission [35]. The authors concluded that in TNF-experienced patients, ustekinumab may, indeed, be more effective in obtaining a clinical response and equally effective in inducing clinical remission [35].

## 4. Case Presentation

We present the case of a 33-year-old male with chronic plaque psoriasis undergoing biologic therapy with secukinumab who developed Crohn’s-like ileitis after ten months of treatment.

**Figure 2:**
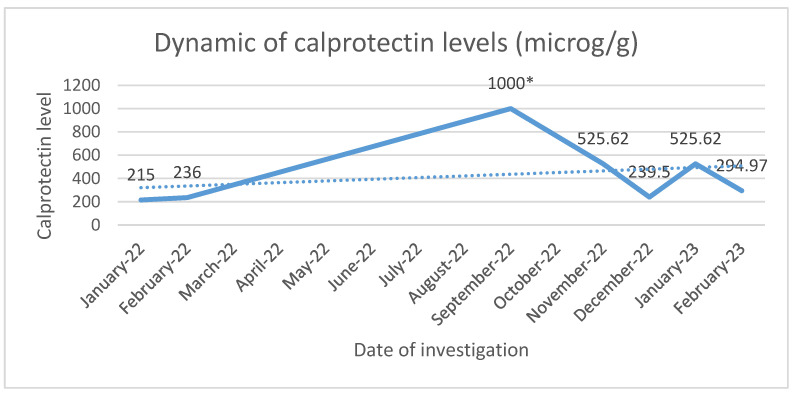
Dynamic of calprotectin levels between January 2022 and February 2023. * >1000 microg/g.

**Figure 3:**
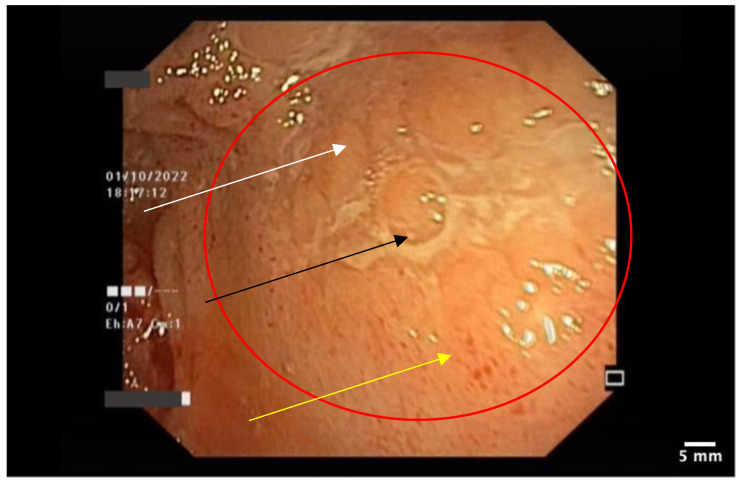
Ileal mucosa with linear ulceration and surrounding erythema. The surrounding area shows edema (white arrow), superficial ulceration (black arrow), and erythema (yellow arrow).

**Figure 4:**
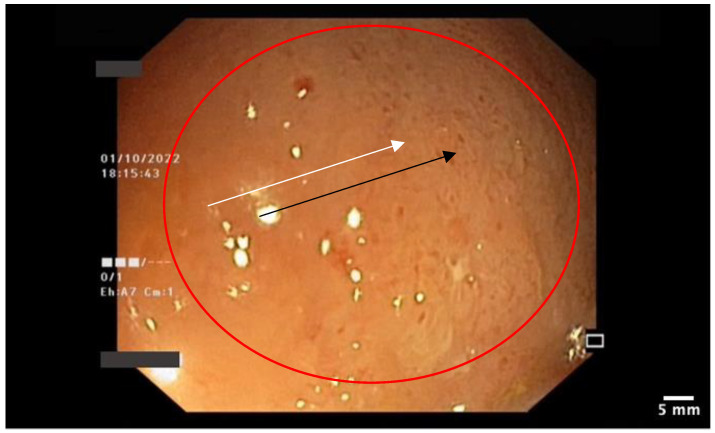
Ileal mucosa: granular mucosa (white arrow) with diffuse erythema (black arrow).

**Figure 5:**
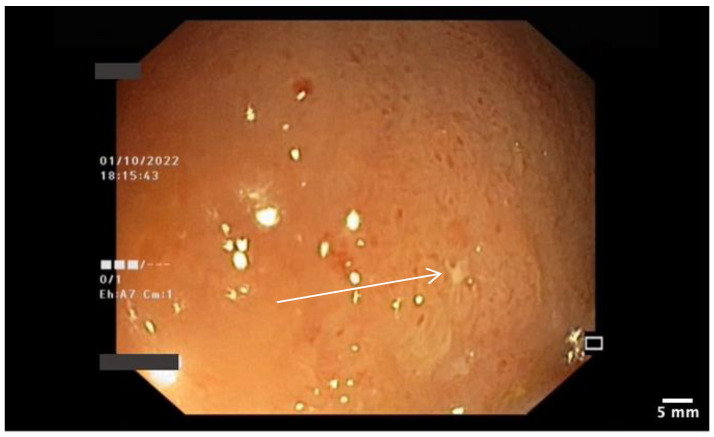
Ileal mucosa: aphthous ulceration (white arrow) and acute edematous changes in the mucosa.

**Figure 6:**
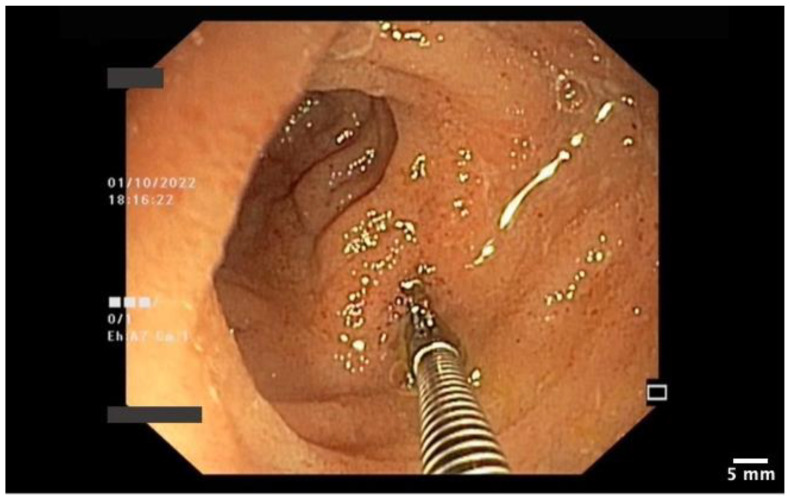
Endoscopic biopsy of the ileal mucosa.

**Figure 7:**
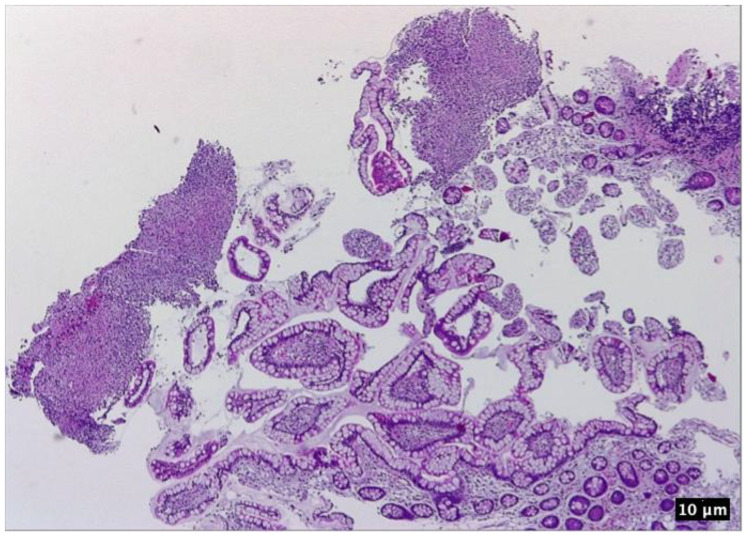
Ileal mucosa. HE 100×. Features of active disease: inflammatory infiltrate consisting in neutrophils, lymphocytes expanding the lamina propria, ulceration, some portions of normal-appearing mucosa; eosinophilic necrotic debris may be seen in the upper left part of the picture.

**Figure 8:**
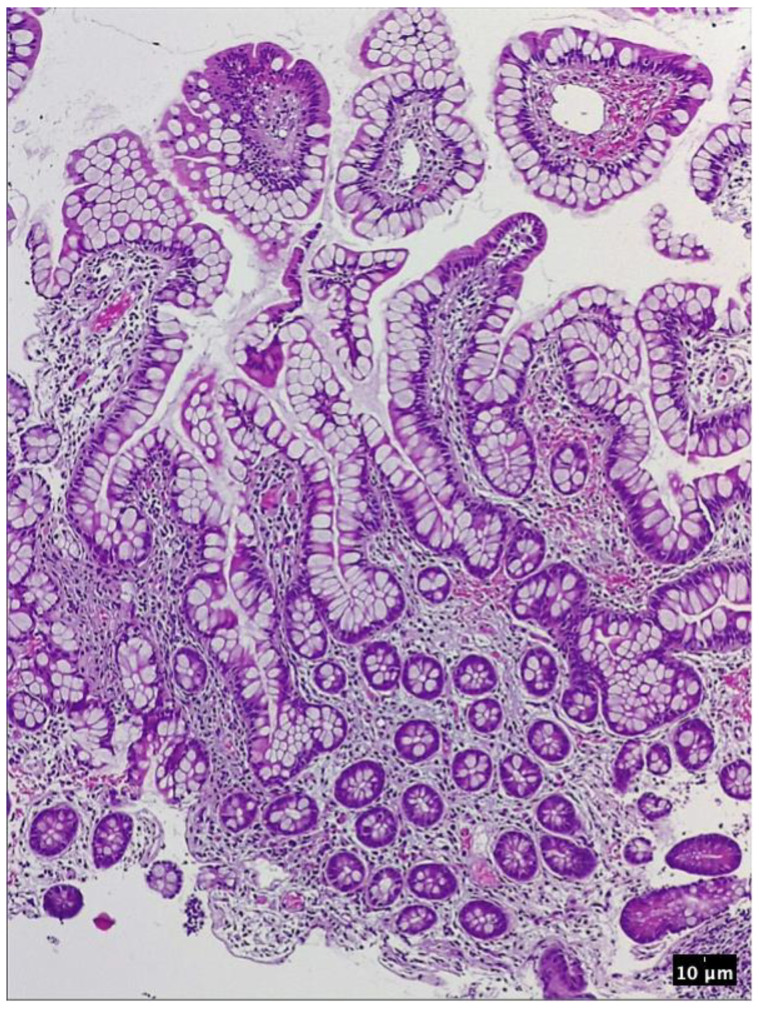
Ileal mucosa. HE 200×. Finger-like projections consisting in a layer of columnar absorbtive cells and goblet cells lining the lamina propria which contains dilated capillaries, as well as marked inflammatory infiltrate and a thin network of fibrin.

**Figure 9:**
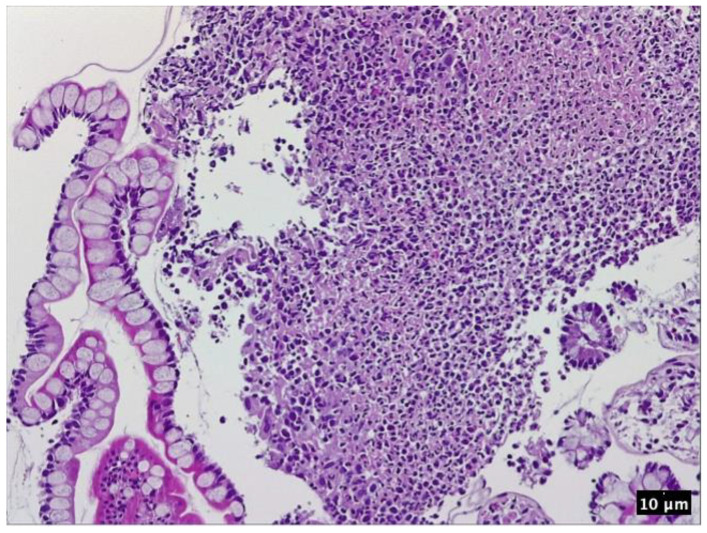
Ileal mucosa—detail. HE 400×. Necrotic debris mixed with fibrin and inflammatory infiltrate consisting in neutrophils and a few lymphocytes in the center and right part of the picture; a layer of columnar and hyperplastic goblet cells may be seen in the left part of the picture.

The patient had a personal history of chronic plaque psoriasis, which was diagnosed in August 2014 and treated with methotrexate 15 mg weekly until 2021, when the medication not only became ineffective, but also led to altered hepatic laboratory tests. During therapy with methotrexate, the patient reported mild, unspecified gastrointestinal symptoms consisting, mainly, in an accelerated intestinal transit. The patient did not have a personal or family history of inflammatory bowel disease; therefore, in November 2021, the patient was initiated on secukinumab, an IL-17 inhibitor. At the 12-week evaluation, there was a significant improvement in both Dermatology Life Quality Index (14 before biologic therapy compared with 0 at the 12-week evaluation) and Psoariasis Area Severity Index (29.6 before biologic therapy compared with 0 at the 12-week evaluation), suggesting an optimal control of the cutaneous disease. At the the six-month evaluation from October 2022, the patient pointed out that he still presented those mild gastrointestinal symptoms, which he correlated with poor dietary habits. A multidisciplinary approach between the Dermatology and Gastroenterology Departments was made for further investigations. Among the laboratory tests performed, there was fecal calprotectin, which, initially, had slightly increased values. Microbiological investigations on a stool sample ruled out infections. At the following evaluation, in the context of a significant increase in the calprotectin levels, as seen in Figure 2, it was decided to perform a colonoscopy with endoscopic biopsies. The colonoscopy revealed multiple ulcerations associated with a pronounced erythema and edema of the perilesional mucosa in the terminal ileum (Figure 3, Figure 4, Figure 5 and Figure 6). Multiple biopsies were taken from the terminal ileum, showing only acute inflammatory changes, suggesting a recent onset and a self-limited course of the disease (Figure 7, Figure 8 and Figure 9).

The gastroenterology report stated that taking into consideration the patient’s personal history of long-standing psoriasis under biologic therapy, as well as the results from the laboratory and endoscopic and histopathologic examinations, the final diagnosis was Crohn’s-like ileitis in the context of biologic therapy with an IL-17 inhibitor. The therapeutic approach consisted in corticosteroid therapy with budesonide, 3 mg orally, three times daily for one month, then two times daily for one month and, finally, once daily for one month, the treatment being withdrawn in February 2023. Under this therapeutic regimen, a proper control of the IBD with clinical remission was obtained.

Because it was established that the gastrointestinal symptoms may have appeared in the context of biologic therapy with secukinumab, a shift in the management of psoriasis had to be performed. Therefore, a collaborative decision between the dermatologist and the gastroenterologist was made. The patient was switched to risankizumab, an IL-23 inhibitor, which has proven to be efficacious in inducing and maintaining clinical remission of inflammatory bowel diseases, while being an excellent therapeutic option for psoriasis, as well. The patient continued follow-up at the Dermatology Department, as well as the Gastroenterology Department.

## 5. Discussion

From a pathopysiological point of view, there are distinct populations of T helper lymphocytes, each with a unique cytokine profile and key roles in both health and disease [50]. For instance, T helper 17 lymphocytes produce IL-17 cytokine subsets which induce both protective and injurious changes in tissue-specific immunity [51,52]. IL-17A is defined as a fundamental mediator of chronic tisular inflammation [52]. Besides IL-17A, Th17 lymphocytes also produce IL-17F, IL-21, and IL-22, which can be either pathological or not, strongly depending upon their transcriptional and functional profile [52]. Under the influence of IL-23, the enhanced expansion of Th-17 cells leads to an excessive IL-17 production which promotes, furthermore, the release of proinflammatory mediators from keratinocytes, epithelial cells, and leukocytes [50,53]. In psoriasis, IL-17A-mediated inflammation contributes to acanthosis and plaque formation; therefore, its blocade via IL-17 inhibitors leads to clinical remission [53]. Cessation of IL-17-mediated inflammation through IL-17 inhibitors was expected to be found, in theory, in inflammatory bowel disease as well; however, nowadays, multiple studies demonstrate the contrary [50,54,55,56]. Experimental studies on mouse models show an exacerbation of colitis following IL-17A or IL-17RA blockade [54,55,56]. In an experimental study from 2015, Maxwell J et al. showed, in a mouse model with colitis, that IL-17A/RA inhibition led to a number of negative consequences: an elevated intestinal barrier permeability, with marked fragility of the intestinal mucosa, an imbalance between effector and regulator CD4+ T lymphocytes, as well as a decreased expression of antimicrobial peptides [54].

In 2023, Deng Z et al. published a paper on the prevalence, clinical features, and management of anti-IL-17 agent-related IBD events [57]. In the study, the authors used data from 2015 to 2022 on anti-IL-17 agents to identify gastrointestinal adverse events and estimated the reporting odds ratios (RORs), as well as corresponding 95% confidence intervals [57]. Moreover, a retrospective analysis of case reports and case series from 2015 to 2022 on inflammatory bowel disease induced by anti-IL-17 agents was developed [57]. A total number of 388 cases of gastrointestinal inflammatory events in the context of IL-17 inhibitor therapy were reported, of which 268 were IBD due to biologic therapy with secukinumab (ROR = 2.13, 95% CI [1.96–2.30]) and ixekizumab (ROR = 2.79, 95% CI [2.39–3.27]) [57]. A total of 29 cases showed solid evidence of IBD after therapy with ixekizumab (20.6%) and secukinumab (79.4%) [57]. The main initial symptoms were diarrhea (90.9%), abdominal pain (57.6%), and bloody diarrhea (51.5%), with 120 colitis cases [57]. Fecal calprotectin was found to be increased in some cases [57]. Complete clinical remission was obtained with corticosteroids and TNF-alpha inhibitors, either in combination or in monotherapy [57]. The authors highlight the importance of obtaining a detailed patient history before initiation of treatment with an IL-17 inhibitor, as well as monitoring for gastrointestinal events during biologic therapy through clinical evaluations and intestinal inflammatory biomarkers [57].

A clinical study from 2012 by Hueber W et al. assessed the influence of secukinumab, a human monoclonal anti-IL-17A antibody on moderate to severe Crohn’s disease [58]. In this randomized, double-blind, placebo-controlled study, 59 patients with moderate to severe Crohn’s disease were enrolled and administered either 2 × 10 mg/kg intravenous secukinumab or placebo [58]. The authors aimed to assess the probability of secukinumab to reduce the Crohn’s Disease Activity Index (CDAI) by more than 50 points by week six [58]. During the study, 14 serious adverse effects occurred in 10 of the subjects (seven secukinumab, three placebo) [58]. Moreover, twenty infections were seen in the secukinumab group, of which four were local fungal infections, versus none in the placebo group [58]. Results showed that not only was the inhibition of the IL-17 axis ineffective with secukinumab, failing to reduce the mean CDAI by >50 points more than placebo at week six, but the therapy was also associated with higher rates of worsening of the gastroenterologic disease and adverse events [58].

We hereafter summarize the existing case reports identified in the literature dealing with a similar clinical scenario, focusing on the management decisions in a case-by-case approach (Table 3).

In 2018, Wang J et al. reported the case of a 41-year-old female who presented at the emergency department with severe abdominal pain, fever, and hourly bloody bowel movements [59]. The patient had previously presented to another hospital with abdominal pain and hematochezia one week after receiving her first dose of secukinumab for psoriasis [59]. Before biologic therapy with an IL-17 inhibitor, the patient had received adalimumab and ustekinumab and had been off immunosuppressive therapy for three months before starting secukinumab [59]. The patient was a non-smoker, with a family history of Crohn’s disease and ulcerative colitis [59]. On the current admission, the patient received methylprednisolone intravenously 40 mg/day. Her C-reactive protein was 128 mg/L, and hemoglobin decreased from 10.6 to 9.7 mg/L [59]. A flexible sigmoidoscopy was carried out and it showed moderate to severe colitis with several profound ulcers in the proximal sigmoid colon [59]. The histopathology report of the endoscopic biopsies revealed the diagnosis of moderately active IBD with colitis [59]. Cefepime and metronidazole were initiated, as well as cyclosporine, 2 mg/kg continuous infusion as salvage therapy [59]. Her fever resolved within two days [59]. She was discharged with oral cyclosporine and a prednisone taper [59]. At follow-up, the patient had an improved clinical condition and she was switched from cyclosporine to infliximab and methotrexate as maintenance therapy, to properly control both psoriasis and the inflammatory bowel disease [59].

In 2018, Philipose J et al. reported the case of a 31-year-old man with a three-week history of abdominal pain and bloody diarrhea accompanied by weight loss, chills, and tenesmus [60]. The only significant aspect in the patient’s medical history was chronic plaque psoriasis treated with ixekizumab, an IL-17 inhibitor for three months prior to his presentation [60]. The patient did not have a family history of inflammatory bowel disease [60]. He reported a ten pack-years smoking history [60]. The patient denied recent travel, high-risk sexual behavior, and any blood transfusions [60]. On presentation, the patient had a marked left lower quadrant pain with no guarding [60]. Laboratory tests showed an increase in white cell count, erythrocyte sedimentation rate of 69 mm/h, and a C-reactive protein of 71.1 mg/L [60]. HIV, ANCA serology, as well as liver profile and hepatitis panel were negative [60]. Stool testing showed only an increase in white cells and cultures were negative for pathogens [60]. The abdominal computed tomography scan showed pancolitis without drainable fluid collection [60]. Flexible colonoscopy showed severe ulcerative proctosigmoiditis [60]. The histology report revealed typical features of new-onset UC [60]. He was administered oral mesalamine 4 g daily, intravenous methylprednisolone 60 mg daily, and parenteral fluids [60]. After two weeks, he developed *C. difficile* infection; therefore, he was given 125 mg vancomycin orally for ten days. Nevertheless, the abdominal pain and bloody diarrhea recurred [60]. He developed anorexia, a decrease in albumin, as well as hemoglobin [60]. Parenteral nutrition was carried out and one unit of packed red blood cells was administered [60]. At this point, flexible sigmoidoscopy showed worsening erosions and deep ulcers [60]. Biopsies from the ulcers were positive for *Cytomegalovirus* (CMV) inclusion bodies [60]. The final diagnosis was steroid-refractory UC with a superimposed CMV infection [60]. He was administered infliximab, 5 mg/kg, and ganciclovir intravenously, 5 mg/kg every twelve hours with significant improvement in his clinical condition after one week [60]. He was discharged on oral valganciclovir for three weeks and infliximab infusions every eight weeks, with optimal control of the IBD [60].

In 2019, Haidari W et al. reported the case of a 65-year-old male with a clinical history of psoriasis and psoriatic arthritis in whom an asymptomatic Crohn’s disease was diagnosed while he was under biologic therapy with secukinumab [61]. The patient had previously been treated with a broad range of medications for psoriasis and psoriatic arthritis: methotrexate, adalimumab, etanercept, and intermittent dexamethasone regimes for joint pain [61]. Nevertheless, each of the aforementioned therapies became, eventually, less efficient, and ultimately the patient was switched to secukinumab, with an outstanding control of both psoriasis and psoriatic arthritis [61]. After a year and a half of biologic therapy with secukinumab, an elective colonoscopy for colorectal cancer screening revealed multiple ulcers and inflammation in the terminal ileum, which were highly suggestive of Crohn’s disease, macroscopically with a normal-appearing colon [61]. Multiple endoscopic biopsies were taken from the terminal ileum [61]. Histological examination of the biopsies from the terminal ileum revealed chronic active ileitis with no crypt abscesses nor granulomas [61]. Evidence of dysplasia or atypia suggesting malignancy were absent as well [61]. Because the patient did not have a history of gastrointestinal symptoms and the only significant aspect in the personal history was a long-standing psoriasis with psoriatic arthritis under with secukinumab, the final diagnosis was asymptomatic CD, probably in the context of biologic therapy with an IL-17 inhibitor [61]. Consequently, biologic therapy with secukinumab was discontinued and the patient was started on ustekinumab, an IL-12/23 inhibitor [61]. Nevertheless, under ustekinumab, psoriasis lesions and arthralgia had recurred; therefore, after a while, the patient was switched to guselkumab, an IL-23 inhibitor, with adequate control of both diseases [61]. However, no follow-up colonoscopy was performed [61].

In 2019, Smith MK et al. reported the case of a 42-year-old Caucasian male who presented with a two-week lower abdominal pain, non-bloody diarrhea accompanied by tenesmus, and nocturnal episodes to the Emergency Department [50]. Other symptoms were represented by fever and chills, persistent in spite of ibuprofen and acetaminophen administration [50]. Laboratory investigations showed an inflammatory syndrome, with an increased C-reactive protein (CRP) of 280 mg/L, while the abdominal CT-scan showed mural thickening of the ascending colon all the way to the distal portion of the descending colon and reactive retroperitoneal lymphadenopathy [50]. Three days later, the patient developed rectal bleeding and the Gastroenterology Department was consulted [50]. There was no history of travelling antibiotic use, sick contacts, nor dietary changes [50]. The medical history of the patient was notable for chronic plaque psoriasis with palmo-plantar involvement [50]. In the last 15 years, the disease had been controlled with acitretin and cyclosporine courses and, finally, with subcutaneous injections of ixekizumab, an IL-17 inhibitor [50]. The gastrointestinal symptoms appeared two days after the twelve-week induction period of ixekizumab [50]. Stool samples for cultures of *C. difficile* toxins, ova and parasites, bacterial pathogens, and fecal leukocytes were negative [50]. Colonoscopy findings, along with the histopathological examination, yielded the diagnosis of Crohn’s-like colitis [50]. The therapeutic approach consisted in prompt cessation of ixekizumab, administration of intravenous corticosteroids, and an escalated TNF-alpha inhibitor therapy, namely infliximab 10 mg/kg at week zero, week one, and week five, and every four weeks for four months [50]. Nevertheless, under infliximab, the chronic plaque psoriasis deteriorated clinically [50]. Following an interdisciplinary collaboration with the patient’s dermatologist, ustekinumab, an IL-12/23 inhibitor, was the biologic agent of choice to ensure therapeutic coverage for both psoriasis and the gastroenterologic illness [50]. From that moment, the patient was asymptomatic in terms of gastrointestinal symptoms and laboratory testing showed no inflammatory syndrome, with a C reactive protein of 4 mg/L [50]. The patient continued follow-up at the Dermatology Department and Gastroenterology Department with careful monitoring of the clinical status, albumin, and C reactive protein [50]. Since psoriasis is associated with an increased risk for inflammatory bowel disease, the authors concluded that regardless of the IL-17 inhibitor therapy, the patient already had an elevated risk for developing CD, compared to the general population [50].

In 2019, Achufusi TD et al. reported the case of a 39-year-old male with a past history of psoriasis under secukinumab, who presented with abdominal pain, fever, chills, and bloody diarrhea [62]. Abominal computed tomography showed increased mural thickness of the descending and sigmoid colon [62]. Consequently, he was administered antibioticotherapy with piperacillin-tazobactam and corticosteroids for a possible autoimmune colitis [62]. On flexible sigmoidoscopy, ulceration of the splenic flexure and moderate to severe active colitis were found, with no signs of inflammation past the transverse colon [62]. Endoscopic biopsies showed architectural disarrangement with shortening and variation in shape and size of the colonic crypts, along with inflammatory infiltrate consisting, mainly, in neutrophils [62]. Because the clinical condition did not improve as expected, the patient was transferred to another medical institution for further investigations [62]. Laboratory evaluation showed marked inflammatory syndrome, with elevated values of C-reactive protein and elevated white blood count [62]. The patient continued to present five to six bloody stools per day, as well as abdominal pain [62]. It was decided to initiate infliximab, along with corticosteroid therapy [62]. A spectacular improvement in the clinical condition was obtained with gradual recovery during the following few weeks [62]. Therapy with secukinumab was ceased and the patient was initiated on apremilast, with proper control of psoriasis [62].

In 2020, Merino Gallego E, Torres Gomez K, and Martinez Amate E presented the case of a 42-year-old male with severe inverse psoriasis under biologic therapy with ixekizumab [63]. The patient had, as well, a personal history of sleep apnea/hypopnea syndrome and smoking [63]. Two weeks after being initiated on ixekizumab, the patient developed fever, abdominal pain, and diarrhea [63]. The patient was admitted to the Gastroenterology Department where further investigations were carried out [63]. Microbiological studies of the feces were negative, as well as the blood cultures [63]. Colonoscopy revealed acute inflammation with aphtoid erosions, fibrinous patchy ulcers from the rectum to the cecum and terminal ileum [63]. Several endoscopic biopsies were taken and the histology report showed cryptitits with cryptic microabscesses, non-necrotizing granulomas, fissuring ulcers, inflammatory infiltrate consisting in neutrophils and lymphocytes, findings attributed to active-phase CD [63]. Therapy with systemic glucocorticoids was started, with successful induction of clinical remission of the intestinal disease [63].

In 2020, Nazarian A, Grin A, Wijeratne DT reported the case of a 48-year-old female who presented to the emergency department with a two-day history of abdominal pain and vomiting [64]. Her medical history consisted in chronic plaque psoriasis diagnosed 15 years before her presentation [64]. The cutaneous disease had been previously treated with calcipotriol and betamethasone ointments, phototherapy, as well as cyclosporine, methotrexate, and finally, ixekizumab, which she was on for 12 weeks before the onset of her gastrointestinal symptoms [64]. The patient had a 15 pack-year smoking history and no familial medical history of inflammatory bowel disease [64]. Laboratory investigations showed elevated white blood cell count, inflammatory syndrome with elevated C-reactive protein and erythrocyte sedimentation rate; microbiological studies of stool samples were negative [64]. The patient underwent abdominal computed tomography, which showed mural thickening of the terminal ileum, as well as the proximal cecum [64]. Subsequently, a colonoscopy was performed, showing punctate ulcerations of the terminal ileum [64]. Histologic examination of the endoscopic biopsies demonstrated acute inflammatory infiltrate with granuloma formation consistent with CD [64]. Following a subsequent literature research, the possible induction of CD by ixekizumab, an IL-17 inhibitor, was taken into consideration [64]. The patient was administered glucocorticoid therapy with budesonide, which led to proper remission of the gastrointestinal symptoms [64]. The patient was also consulted by the Rheumatology Department and diagnosed with psoriatic arthritis [64]. A multidisciplinary decision-making process between the Gastroenetrology, Dermatology, and Rheumatology Departments was made regarding further therapeutic approach; therefore, the patient was switched to adalimumab, which successfully controlled psoriasis, CD, and psoriatic arthritis [64].

In 2021, Marin M et al. reported the case of a case of a 76-year-old Spanish woman who presented with acute self-limited colitis after receiving ten doses of ixekizumab for her pre-existing, long-standing chronic plaque psoriasis [65]. She had previously been treated with methotrexate 20 mg, acitretin 25 mg, both of them being withdrawn due to intolerance, and adalimumab 40 mg subcutaneously for two years [65]. The patient was then switched to ustekinumab 45 mg every 12 weeks due to loss of therapeutic response to the TNF-alpha inhibitor [65]. Nevertheless, after five doses of ustekinumab, the patient developed bullous pemphigoid, possibly in the context of biologic therapy with the IL-12/23 inhibitor [65]. The patient was then switched to ixekizumab, 80 mg subcutaneously every 4 weeks after a previous induction regimen [65]. After ten doses of ixekizumab, the patient presented with bloody diarrhea for two weeks and occasional abdominal pain accompanied by fever and weight loss [65]. The patient had no family history of inflammatory bowel disease [65]. Microbiological examination of blood culture, *Clostridium difficile* toxin determination, and stool culture were negative [65]. Colonoscopy examination was compatible, macroscopically, with inflammatory colitis of the left colon [65]. Since ixekizumab was the major suspicious drug for this gastrointestinal adverse event, the discontinuation of the biologic therapy with the IL-17 inhibitor was proposed [65]. Intravenous methylprednisolone (1 mg/kg/day) was initiated and the patient was discharged with a prescription of prednisone, 30 mg daily, orally, tapered over a 30-day course [65]. After ixekizumab withdrawal, the gastrointestinal symptoms remitted [65]. The patient was successfully switched to guselkumab, 100 mg subcutaneously, every eight weeks for the management of chronic psoriasis, with an adequate response [65].

In 2021, Mu X et al. reported the case of a new-onset drug-associated colitis in a 45-year-old male receiving ixekizumab for chronic plaque psoriasis [66]. The patient had a three-week history of over 10 episodes of diarrhea per day, diffuse abdominal pain, and tenesmus, with no other systemic signs or symptoms [66]. The only significant aspect in the personal history of the patient was the long-standing chronic plaque psoriasis for which he was receiving ixekizumab [66]. The patient underwent therapy with multiple biologic agents, but significant control of the disease was obtained when the patient was switched to ixekizumab, nine months before the onset of the gastrointestinal symptoms [66]. Moreover, the patient did not have a family history of colorectal cancer or inflammatory bowel disease [66]. The patient was admitted to the internal medicine department and underwent further investigations [66]. The flexible sigmoidoscopy showed punched-out ulcerations in the left colon alternating with regions of normal mucosa [66]. An endoscopic biopsy was performed [66]. The pathology report demonstrated histopathologic features consistent with Crohn’s disease [66]. High-dose corticosteroid therapy was initiated; nevertheless, two days later, the patients developed toxic megacolon, which was complicated by perforated viscus, as shown by the intraperitoneal free air on the abdominal X-ray [66]. The patient underwent total colectomy and, due to persistent intra-abdominal sepsis and consequently, ongoing small bowel ischemia, the patient underwent resection of the distal ileum [66]. Rehabilitation in the surgery ward was required for an additional two-week period, before the patient was finally discharged with repeated follow-ups to the general surgery and gastroenterology department [66]. Nine months post-discharge, the patient presented with a favorable recovery [66]. Because the patient had no family history of inflammatory bowel disease, the presentation was atypical and the only significant aspect was the concomitant chronic plaque psoriasis controlled with an IL-17 inhibitor; the final diagnosis was drug-associated colitis in the context of biologic therapy with ixekizumab [66]. After cessation of ixekizumab, the patient did not present recurrence of the clinical gastrointestinal disease [66].

## 6. Conclusions

In recent years, the use of IL-17 inhibitors has become more and more prevalent against psoriatic disease. Nevertheless, in some patients, this biologic class may possibly play an important part in inducing inflammatory bowel disease. This review and case presentation aids in increasing awareness of the importance of closely monitoring psoriasis patients under IL-17 inhibitors for the appearance of gastrointestinal symptoms, in order to adequately adjust their biologic therapy if required.

## Figures and Tables

**Table 1:** Available novel therapeutic options for moderate to severe psoriasis and mechanism of action.

Systemic Treatment (Biologic Therapy Class and Novel Small-Molecule Inhibitor)	Drug	Chemical Structure	Mechanism of Action
IL-17 inhibitors	Ixekizumab	Humanized immunoglobulin G4 monoclonal antibody	Binding to interleukin 17 (IL-17A) and blocking its proinflammatory effects.
	Secukinumab	Human IgG1 monoclonal antibody	
IL-23 inhibitors	Guselkumab	Recombined human monoclonal antibody	Selective binding and blocking the p19 alpha subunit of IL-23.
	Risankizumab	Humanized IgG monoclonal antibody	
	Tildrakizumab	Recombined human monoclonal antibody	
IL-12/23 inhibitors	Ustekinumab	Human monoclonal antibody	Binds to the p40 subunit common to IL-12 and IL-23, thus preventing their interaction with the IL-12 receptor β1 subunit of the IL-12 and IL-23 receptor complexes.
TNF-alpha inhibitors	Adalimumab	Human monoclonal antibody	Binds with specificity to tumor necrosis factor-alpha (TNF-alpha) and inhibits its interaction with cell receptors.
	Infliximab	Chimeric monoclonal antibody	
	Etanercept	Fusion protein produced by recombinant DNA	
	Certolizumab pegol	PEGylated, humanized, antigen-binding fragment of an anti-TNF monoclonal antibody	
Small-molecule inhibitor	Apremilast	Polyaromatic molecule, a small-molecule inhibitor of the enzyme phosphodiesterase 4 (PDE4)	It selectively targets and inhibits the activity of small molecules.

**Table 2:** Recommended therapeutic options for concomitant diseases in patients with chronic plaque psoriasis.

Comorbidity	Recommended Therapeutic Option
Psoriatic arthritis	TNF-α inhibitors, ixekizumab, secukinumab, guselkumab, risankizumab
Congestive heart failure	IL-17 and IL-23 inhibitors
Metabolic syndrome	IL-17 inhibitors, ustekinumab, IL-23 inhibitors, apremilast
Depression	IL-23 inhibitors
Malignancy	IL-17 inhibitors, IL-23 inhibitors, apremilast
Inflammatory bowel disease	TNF-alpha inhibitors (adalimumab, infliximab, certolizumab pegol), ustekinumab, IL-23 inhibitors

**Table 3:** Case presentations of IBD associated with IL-17 inhibitors for psoriasis reported in the literature.

Reference	Age and Gender	Type of IL-17 Inhibitor	Duration of Biologic Therapy	Personal or Family History	Gastroenterologic Diagnosis	Management
Wang J et al., 2018 [59]	41-year-old female	Secukinumab	One week	Family history of CD and UC	Moderately active colitis	Corticosteroid therapy Infliximab Methotrexate
Philipose J et al., 2018 [60]	31-year-old male	Ixekizumab	Three months	Smoking history	Steroid-refractory UC with a superimposed Cytomegalovirus infection	Infliximab Antiviral therapy
Haidari W et al., 2019 [61]	65-year-old male	Secukinumab	A year and a half	Psoriatic arthritis	Asymptomatic CD	Switch to Guselkumab
Smith MK et al., 2019 [50]	42-year-old male	Ixekizumab	Twelve weeks	None	Crohn’s-like colitis	Glucocorticoids Ustekinumab
Achufusi TG et al., 2019 [62]	39-year-old male	Secukinumab	Six months	None	Unspecified colitis	Infliximab and glucocorticoids Apremilast
Merino Gallego E et al., 2020 [63]	42-year-old-male	Ixekizumab	Two weeks	None	CD	Corticosteroid therapy
Nazarian A et al., 2020 [64]	48-year-old female	Ixekizumab	Twelve weeks	Smoking history Psoriatic arthritis	CD	Corticosteroid therapy Adalimumab
Marin M et al., 2021 [65]	76-year-old female	Ixekizumab	Twenty weeks	None	Unspecified colitis	Corticosteroid therapy Guselkumab
Mu X et al., 2021 [66]	45-year-old male	Ixekizumab	Nine months	None	CD	Corticosteroid therapy

## Data Availability

Not applicable.

## Abbreviations

HLA	Human leukocyte antigen
DNA	Dezoxyribonulceic acid
TNF-alpha	Tumor necrosis factor-alpha
IL	Interleukin
VEGF	Vascular endothelial growth factor
PDE4	Phosphodiesterase 4
CD	Crohn’s disease
UC	Ulcerative colitis
IBD	Inflammatory bowel disease
OR	Odds ratio
GHAS	Global histology activity scores
mcg	microgram
HE	Hematoxilin and eosin
ANCA	Anti-neutrophil cytoplasmic antibodies
